# ITC-net-audio-5: an audio streaming dataset for application identification in network traffic classification

**DOI:** 10.1186/s13104-024-06718-7

**Published:** 2024-02-27

**Authors:** Mohammad Nikbakht, Mehdi Teimouri

**Affiliations:** Information Theory and Coding (ITC) Laboratory, University of Tehran, Tehran, Iran

**Keywords:** Network Traffic classification, Application identification, Traffic capturing, Audio Streaming, Dataset

## Abstract

**Objectives:**

An essential aspect of network traffic classification is application identification. This involves capturing and analyzing the traffic patterns of applications. There are a few publicly available datasets that specifically capture streaming data from network-based applications. Therefore, our objective is to generate an up-to-date dataset with a focus on audio streaming data. This dataset can be a valuable resource for identifying audio streaming applications in the field of network traffic classification.

**Data description:**

The dataset contains network traffic captured during audio streaming communications on five trending applications: Google Meet, Skype, Telegram, WhatsApp, and SoundCloud. It includes 500 files in PCAP format captured by Wireshark and PCAPdroid tools during voice calls and online music playback. The concurrent utilization of these tools facilitates the avoidance of capturing background traffic.

## Objective

An essential aspect of network traffic classification is identifying applications used within the network. However, this task can be challenging due to the limited availability of datasets [1–3]. To advance this field, it is crucial to provide comprehensive and up-to-date datasets. This data article presents a dataset of network traffic captured during audio streaming communications on five trending applications. Our dataset specifically focuses on audio streaming transmission, which has become increasingly prevalent. Audio streaming is now much more popular than downloading it, and millions of users around the world use applications such as online music and podcast players, voice calling, and other related activities [4, 5]. On the other hand, less attention has been given to generating a dataset that specifically captures audio streaming data from network-based applications.

Given that each network-based application uses its own communication protocol to transmit audio data, it is possible to identify audio streaming applications by analyzing the traffic patterns associated with each one. To do this, researchers can implement their machine learning or deep learning methods on our dataset.

Our dataset is up-to-date and contains the latest trending applications with audio streaming capabilities. This dataset is diverse and includes audio calls and online music playback. The data is precious due to its unique collection method, which prevents capturing background traffic.

This dataset can be a valuable resource for experts in the field of network traffic classification, especially in identifying audio streaming applications. Its applications extend beyond the academic sphere, as it can also be helpful for various companies and research sectors, enabling them to analyze and enhance communication networks.

## Data description

The dataset specifically contains audio streaming data from five applications. It includes 500 PCAP files (i.e., 100 files per application). The dataset is stored at figshare [6]. Each application’s data is stored in a dedicated ZIP file with the application’s name, and they are named as [application name]_[file number].pcap (see Dataset 1 to 5 in Table 1). Alongside the dataset, there is a PDF document containing tables and figures that provide a brief description of the dataset specifications, a visual representation of the methodology, and versions of the tools used (see Data file 1 in Table 1).

The data generation process involved several key stages. More details about each stage will be provided in the following sections:

### Capture tools selection

We utilized Wireshark and PCAPdroid, two effective tools that employ PCAP files to archive network packets [7, 8]. Wireshark captures data from a selected network but is unable to filter specific applications, resulting in the capture of irrelevant background traffic. To address this issue, we employed PCAPdroid, which can capture traffic from selected applications on the source device. However, it should be noted that PCAPdroid modifies the 3 and 4 network layers of the packets [8]. Therefore, for greater assurance, we simultaneously captured network traffic using both Wireshark and PCAPdroid and selected only the packets that were identical in both captures as the final data. These identical packets are essential and do not contain background traffic [9].

### Traffic capture setup

To capture traffic using both Wireshark and PCAPdroid simultaneously, we connected them to the same network. Wireshark is installed on a Windows PC, and PCAPdroid is installed on an Android tablet. The tablet served as the source device. We shared the internet network through the Windows PC, and the tablet connected to it. By starting the capture, Wireshark on PC captured the entire network data, while PCAPdroid on the source device (tablet) simultaneously captured the application data. We used a Python code to filter out non-identical packets from the generated PCAP files [10].

### Applications selection

Five trending applications with audio streaming capabilities were selected: Google Meet, Skype, Telegram, WhatsApp, and SoundCloud. Google Meet, Skype, Telegram, and WhatsApp were used for voice calls, and SoundCloud was used for online music playback. Both the source and destination devices transmitted audio during voice calls. Various music tracks were played to capture online music traffic. We collected 100 data samples, each lasting 3 to 4 min, for each application, resulting in a total dataset of 500 captured data in PCAP format.

### VPNs

Due to internet restrictions in Iran, several applications cannot access their servers. We used VPNs to bypass these restrictions. The following VPN applications were installed on the Windows PC and shared via the local network adapter: TurboVPN (OpenConnect) v8.3.5, Proton VPN 2.3.2, and Windscribe VPN v2.5.18. More details are given in Data file 1 in Table 1. To ensure that packets were not modified by the VPNs, we captured network traffic through the local area network instead of directly from the VPNs. For increased result confidence, some applications were captured using two different VPNs (for more information, see Data file 1 in Table 1).

**Table 1:** Overview of datasets/data files

Label	Name of dataset/data file	File types (file extension)	Data repository and identifier (DOI number)
Dataset 1	Google Meet	Archive file (.zip) containing packet capture (.pcap) files	figshare (10.6084/m9.figshare.24721035)
Dataset 2	Skype	Archive file (.zip) containing packet capture (.pcap) files	figshare (10.6084/m9.figshare.24721035)
Dataset 3	Telegram	Archive file (.zip) containing packet capture (.pcap) files	figshare (10.6084/m9.figshare.24721035)
Dataset 4	WhatsApp	Archive file (.zip) containing packet capture (.pcap) files	figshare (10.6084/m9.figshare.24721035)
Dataset 5	SoundCloud	Archive file (.zip) containing packet capture (.pcap) files	figshare (10.6084/m9.figshare.24721035)
Data file 1	Readme	Portable document format (.pdf )	figshare (10.6084/m9.figshare.24721035)

### Limitations

- Applications: Among all audio streaming applications, only five trending applications were selected for capturing their traffic.
- Quantity: Only 100 data samples were collected from each application.
- Time: Each file was approximately 3 to 4 min long.
- Devices: For data collection, only one source device (tablet) and one destination device (mobile) were used.
- Location: The data collection took place in a specific area (University of Tehran, Tehran, Iran).

## Data Availability

The data described in this Data note can be freely and openly accessed on figshare at https://doi.org/10.6084/m9.figshare.24721035. Please see table 1 and references [6] for details and links to the data.

## Abbreviations

PCAP: packet capture
PDF: portable document format
PC: personal computer
VPN: virtual private network
